# Minimizing error in estimates of the effect of interventions by accounting for baseline measurements: A simulation study analyzing effects on child growth

**DOI:** 10.1111/mcn.13547

**Published:** 2023-07-13

**Authors:** Emily L. Deichsel, Kirkby D. Tickell, Elizabeth T. Rogawski McQuade

**Affiliations:** ^1^ Center for Vaccine Development and Global Health University of Maryland School of Medicine Baltimore Maryland USA; ^2^ Department of Global Health University of Washington Seattle Washington USA; ^3^ Childhood Acute Illness & Nutrition (CHAIN) Network Nairobi Kenya; ^4^ Department of Epidemiology Emory University Atlanta Georgia USA

**Keywords:** baseline imbalance, bias, linear growth, power, randomized controlled trial, statistical analysis

## Abstract

Interventions to reduce childhood stunting burden require clinical trials with a primary outcome of linear growth. When growth is measured longitudinally, there are several options for including baseline measurements in the analysis. This study compares the performance of several methods. Randomized controlled trials evaluating a hypothetical intervention to improve length‐for‐age *z*‐score (LAZ) from birth through 24 months of age were simulated. The intervention effect was evaluated using linear regression and five methods for handling baseline measurements: comparing final measurements only (FINAL), comparing final measurement adjusted for baseline (ADJUST), comparing the change in the measurement over time (DELTA), adjusting for baseline when comparing the changes over time (DELTA+ADJUST) and adjusting for baseline in two‐step residuals approach (RESIDUALS). We calculated bias, precision and power of each method for scenarios with and without a baseline imbalance in LAZ. Using a 0.15 effect size at 18 months, FINAL and DELTA required 1200 and 1500 enroled participants, respectively, to reach 80% power, whereas ADJUST, DELTA+ADJUST and RESIDUALS only required 900 participants. The adjusted models also produced unbiased estimates when there was a baseline imbalance, whereas the FINAL and DELTA methods produced biased estimates, as large as 0.07 lower and higher, respectively, than the true effect. Adjusted methods required smaller sample size and produced more precise results than both DELTA and FINAL methods in all test scenarios. If randomization fails, and there is an imbalance in LAZ at baseline, DELTA and FINAL methods can produce biased estimates, but adjusted models remain unbiased. These results warn against using the FINAL or DELTA methods.

## Key messages

- Several options exist for including baseline measurements in the analysis of a randomized control trial measuring linear growth over time.
- Adjusted methods required a smaller sample size and produced more precise results than both DELTA and FINAL methods in all tested scenarios.
- If randomization fails, and there is in imbalance in length‐for‐age *z*‐score at baseline, DELTA and FINAL methods can produce biased estimates, but adjusted models remain unbiased.
- Researchers should avoid using the FINAL or DELTA methods.

## Graphical abstract

When measuring linear growth longitudinally in a randomized clinical trial, analytic methods that adjust for baseline (ADJUST, DELTA+ADJUST and RESIDUALS) require smaller sample size and produced more precise results than using comparing in measurement over time between two groups (DELTA) or comparing the final measurements only (FINAL).

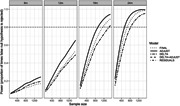

## 1 INTRODUCTION

Linear growth faltering, an abnormally slow rate of gain in a child's height or length, is an important indicator of poor childhood health affecting millions of children globally (Keats et al., 2021). Reduced linear growth during early childhood has been linked to suboptimal cognitive and educational attainment, and increased risk for disease morbidity and mortality (Adair et al., 2013; Cheung & Ashorn, 2010; de Onis & Branca, 2016; Scharf et al., 2018). Available interventions to ameliorate the burden of poor linear growth have had limited effectiveness, with randomized controlled trials often estimating magnitudes of effect that are not considered clinically meaningful (Ahmed et al., 2021; Keats et al., 2021; Pickering et al., 2019). As new interventions to tackle the burden of linear growth faltering continue to be developed, there is a need to optimize the analysis methods used in clinical trials of these novel managements.

Randomized controlled trials targeting linear growth faltering generally use length‐for‐age *z*‐score (LAZ), an age standardized measure of child length, as the primary endpoint. As length can be measured longitudinally, pre‐intervention measurements of the outcome are often obtained. Recent trials serve as examples where length measurements were taken at baseline before any intervention was given and again at follow‐up timepoints (Deboer et al., 2018; Iannotti et al., 2017; Mangani et al., 2015). Consider the Early Life Interventions of Childhood Growth and Development in Tanzania (ELICIT) (Deboer et al., 2018), which randomized antimicrobials and/or nicotinamide to promote growth through 18 months of age. Infants were enroled within 14 days after birth and received study medication periodically from enrolment to 18 months of age. Length was measured at enrolment and every 3 months thereafter. Like other clinical trials, the ELICIT investigators had to define the analysis of length in an a priori statistical analysis plan and specifically how to handle baseline measurements in their primary analysis.

Traditionally, randomized trials do not adjust for baseline covariates, because the randomization process should have balanced both measured and unmeasured covariates across the trial's arms. However, outcomes that can be measured at baseline and longitudinally, such as LAZ, may benefit from the inclusion of the baseline measurement in the trial's primary analysis, as it can correct for chance imbalances across trial arms, improve the precision of effect estimates and improve power to detect a statistically significant effect (Egbewale et al., 2014; Frison & Pocock, 1992; Fu & Holmer, 2015; Kahan et al., 2014; Senn, 1994, 2006; Van Breukelen, 2006; Vickers, 2001; Vickers & Altman, 2001).

There are several methods for incorporating baseline measurements in analyses. Common strategies are to either specify the outcome as the change in LAZ (ΔLAZ from baseline) or to include the baseline measurement as an adjustment variable in the final regression model. Others promote adjusting for baseline using a less common residuals method, first regressing final LAZ on baseline and then comparing the model residuals between intervention groups (Esrey et al., 1990). The relative merits of each analysis approach are described in the statistical literature (Egbewale et al., 2014; Fu & Holmer, 2015; Senn, 2006; Van Breukelen, 2006) and clinical trial guidelines (Comittee for Medical Products for Human Use, 2015), but these papers are not specific to childhood growth, are rarely grounded in real world data and can be somewhat esoteric. This manuscript compares the performance of the methods below and offers recommendations for clinical trialists designing linear growth studies. We illustrate the strengths and weaknesses of the common approaches to modelling linear growth in randomized controlled trials, specifically methods that:

1. Ignore baseline measurements and only compare the endpoint measurement between the two groups (FINAL).
2. Compare the endpoint measurement between the intervention and control groups and adjust for the baseline measurement in a linear regression model (ADJUST).
3. Calculate change from baseline to endpoint for each participant and then compare that delta measurement between the two groups (DELTA).
4. Calculate change from baseline to endpoint for each participant, compare that delta measurement between the two groups but also adjust for the baseline measurement (DELTA+ADJUST).
5. Adjust for baseline by first regressing final LAZ by baseline LAZ and then compare the model residuals between two intervention groups (RESIDUALS).

## 2 METHODS

We used population anthropometric characteristics from The Etiology, Risk Factors and Interactions of Enteric Infections and Malnutrition and the Consequences for Child Health and Development Enteric (MAL‐ED) cohort study Bangladesh site (Acosta et al., 2014) to inform simulations of two‐arm randomized controlled trials. MAL‐ED was a multisite observational birth cohort study that investigated the relationship between malnutrition and intestinal infections and their effects on child growth and development in the global south. Enroled children from eight sites were closely monitored twice weekly for illnesses and monthly anthropometry from their first month of life through 2 years of age. In the present study, we compared multiple approaches to assessing the effect of an intervention randomized at birth on linear growth at 6, 12, 18 and 24 months of age. We estimated the effect of the intervention using linear regression and compared the power, bias and precision of FINAL, ADJUST, DELTA, DELTA+ADJUST and RESIDUALS methods at varying sample sizes and various correlations between baseline and follow up measurements. Finally, we ran these simulations under scenarios with and without an imbalance in baseline LAZ.

### 2.1 Experimental conditions

The outcome of the trial was LAZ measured at baseline/birth, 6, 12, 18 and 24 months of age. The mean LAZ and standard deviation at each time point, as well as the correlation between measurements across time points, were selected from normal distributions based on data collected in the MAL‐ED Bangladesh (Acosta et al., 2014) and are summarized in Table 1. The intervention effect was simulated as an increase in LAZ by 0.05 each 6 months of life, that is, the cumulative effect was 0.05 at 6 months, 0.10 at 12 months, 0.15 at 18 months and 0.20 at 24 months. The final effect of 0.20 at 24 months of age was based on results from Sanitation Hygiene Infant Nutrition Efficacy (SHINE) trial (Pickering et al., 2019).

**Table 1:** Simulated LAZ at each time point and correlation parameters for LAZ between timepoints.

Time point (months)	Correlation					LAZ mean (SD)
	0	6	12	18	24	
0	1					−1.10 (1.03 SD)
6	0.61	1				−1.19 (0.97 SD)
12	0.47	0.87	1			−1.66 (0.93 SD)
18	0.46	0.80	0.9	1		−1.95 (0.93 SD)
24	0.44	0.76	0.88	0.95	1	−2.03 (0.94 SD)

Abbreviations: LAZ, length‐for‐age *z*‐score. SD, standard deviation.

We simulated two scenarios considering the balance of LAZ at baseline. An imbalance across randomized intervention arms in factors measured at baseline that are strongly related to the outcome can cause bias in the intervention effect estimate. In the first scenario, LAZ at birth was balanced between the randomization arms (Balanced Scenario), meaning key variables have approximately the same distribution in both randomization arms. In the second scenario, there was an imbalance in the baseline value (Imbalanced Scenario), meaning the distribution of key variables is not the same between the two randomization groups. We created these baseline distributions of LAZ (balanced and unbalanced) by either randomly allocating participants to the two groups with equal probability or by making the group allocation probability dependent on the baseline LAZ measurement. Specifically, the Balanced Scenario used a 1:1 allocation ratio (probability of being in the intervention group was 0.5) to assign the intervention arm. In comparison, group assignment for the Imbalanced Scenario depended on the baseline LAZ (probably of being in the intervention group was 0.47–0.03 × baseline LAZ), simulating a scenario where children with lower LAZ at baseline are more likely to be randomized to an intervention group. The target mean baseline imbalance was 0.12 LAZ. We calculated this target using half of the difference in enrolment LAZ (0.22 LAZ) between children of ‘high’ and ‘low’ socioeconomic status. High and low socioeconomic status was defined by the continuous water/sanitation, assets, maternal education and household income score that was used in MAL‐ED (Psaki et al., 2014). The mean baseline imbalance in LAZ and percent of trials with a statistically significant imbalance (*p* < −0.05 as tested when regressing baseline LAZ by intervention group) for each sample size is included in Supporting Information: Table S1.

The intervention effect in each simulated trial was estimated by the regression coefficient (*β*_1_) corresponding to the intervention group variable (*x*) in the linear regression models below. The regression equations for each statistical method are shown below for each timepoint *i* (i.e., 6, 12, 18, 24 months).

ADJUST:

(1) $${\mathrm{LAZ}}_{i}=\beta_{0}+\beta_{1}x+\beta_{2}{\mathrm{LAZ}}_{\mathrm{birth}}$$

DELTA:

(2) $${\mathrm{LAZ}}_{i}-{\mathrm{LAZ}}_{\mathrm{birth}}=\beta_{0}+\beta_{1}x$$

DELTA+ADJUST:

(3) $${\mathrm{LAZ}}_{i}-{\mathrm{LAZ}}_{\mathrm{birth}}=\beta_{0}+\beta_{1}x+\beta_{2}{\mathrm{LAZ}}_{\mathrm{birth}}$$

FINAL:

(4) $${\mathrm{LAZ}}_{i}=\beta_{0}+\beta_{1}x$$

RESIDUALS:

(5a) $${\mathrm{LAZ}}_{i}=\beta_{0}+\beta_{2}{\mathrm{LAZ}}_{\mathrm{birth}}$$

(5b) $$\mathrm{Residual}\left(5a\right)=\beta_{3}+\beta_{1}x.$$

For both scenarios, the clinical trial was simulated with 15 different sample sizes, 100–1500 by increments of 100. Each of the 30 unique trial scenarios (2 scenarios × 15 sample sizes) was simulated 1000 times to generate robust estimates. Within each simulated trial, we applied the five statistical approaches to estimating effects on LAZ outcomes at four postrandomization timepoints. Detailed information about the simulation methods is included in the Supporting Information: Materials.

### 2.2 Assessing model performance

For each trial analysis, we calculated the median effect estimate (the median regression coefficient for each scenario). The 95% uncertainty intervals (95% UIs) represented the 2.5th and the 97.5th percentile of the effect estimates from the 1000 trials.

To measure bias in the intervention effect estimated by the five statistical approaches, we calculated the difference between the effect estimate (median regression coefficient) and the true intervention effect for a specific timepoint. Forest plots by method and time point displayed the median intervention effect estimate and 95% UI for simulated studies with sample size equal to 1000.

The power of each method was calculated as the proportion of trials for which the null hypothesis was correctly rejected within each unique scenario, sample size and timepoint. We displayed the relationship of calculated power with sample size and correlation between baseline and endline LAZ using a scatterplot with locally weighted scatterplot smoothing locally weighted scatterplot smoothing curve. We extended the simulation described above for the Balanced Scenario to sample sizes of 12,000 children to estimate the number of participants, rounded to nearest 100 children, required to reach 80% power for each analysis and timepoint. Formulas exist to calculate samples sizes by analytic method in place of simulations (Clifton et al., 2019).

To quantify the precision of the five analytic methods, we calculated the mean standard error (SE) of the estimated intervention effect for each simulated trial analysis. We also calculated coverage as the proportion of 95% confidence intervals for the estimated intervention effect from each of the 1000 simulated trials that contained the true intervention effect.

### 2.3 Patient and public involvement

There was no patient and public involvement in this simulation study.

## 3 RESULTS

### 3.1 Balanced Scenario: Trials with no baseline imbalance

In the Balanced Scenario, because there was no systematic imbalance in baseline LAZ between groups, all five analytic methods provided unbiased estimates of the intervention effect at all timepoints (Supporting Information: Table S2). Coverage was also close to 95% as expected for all time points and we did not observe any clear trend in bias across methods within this study's simulation parameters (Supporting Information: Figure S1).

**Figure 1:**
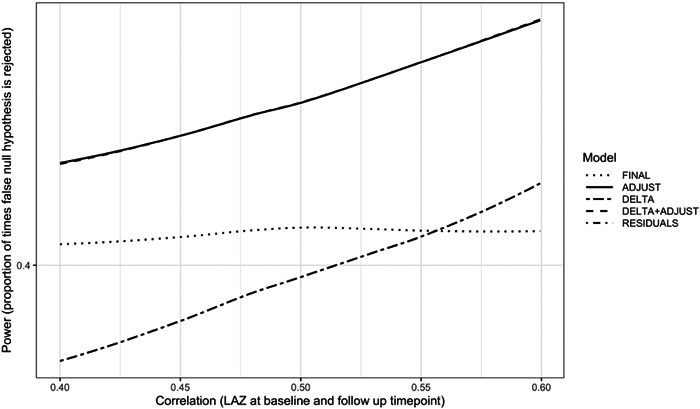
Balanced Scenario: Power (the proportion of trials for which the null hypothesis was correctly rejected) of each modelling method by correlation between baseline and endline length‐for‐age *z*‐score (LAZ) when sample size is 1000. Higher power indicates an increased ability to detect a true effect, when such effect exists. Adjusting for baseline when comparing the changes over time (DELTA+ADJUST) model results are identical to adjusted for baseline (ADJUST) and adjusting for baseline in two‐step residuals approach (RESIDUALS) model result and so lines overlap. FINAL, final measurements only.

**Figure 2:**
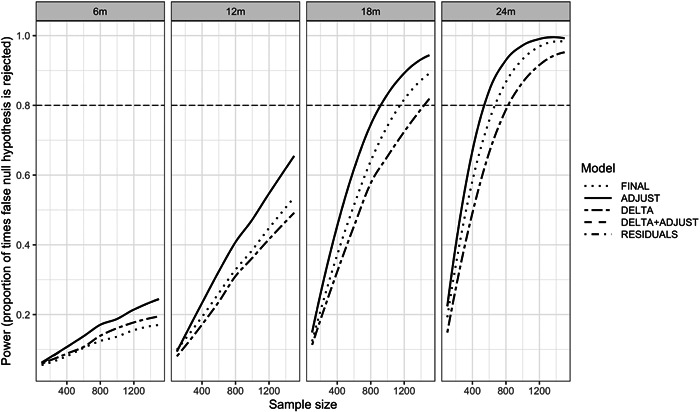
Balanced Scenario: Power (the proportion of trials for which the null hypothesis was correctly rejected) of each modelling method at each time point by sample size (locally weighted scatterplot smoothing [lowess]). Higher power indicates an increased ability to detect a true effect, when such effect exists. Adjusting for baseline when comparing the changes over time (DELTA + ADJUST) and adjusting for baseline in two‐step residuals approach (RESIDUALS) model results are identical to adjusted for baseline (ADJUST) model result and so lines overlap. FINAL, final measurements only.

However, precision and power differed across analysis methods. Methods adjusting for baseline, ADJUST, DELTA+ADJUST and RESIDUALS, offered the most power at all time points and sample sizes relative to the other two methods. The power when using DELTA was equal to or greater than FINAL only at the 6‐month timepoint, where the correlation between baseline and timepoint LAZ was the highest. For all other time points (12, 18 and 24 months), where correlation was <0.5, the power for FINAL was greater than DELTA (Figure 1). The sample sizes required to reach 80% power also varied substantially across the analysis methods. For example, a trial with the outcome measured at the 18‐month timepoint (Figure 2) that planned to use a FINAL LAZ analysis, would require enroling ~33% (1200 vs. 900) more participants than a trial using ADJUST, DELTA+ADJUST or RESIDUALS. Similarly, employing a DELTA LAZ analysis would require approximately 66% (1500 vs. 900) more participants than the three optimal methods under this scenario (Supporting Information: Table S3).

For FINAL, the mean SE was unaffected by changes in correlation and intervention effect across the timepoints. This reflects the fact that FINAL does not take into account any baseline information. For the other methods, DELTA, ADJUST, DELTA+ADJUST and RESIDUALS, we observed a small increase in mean SE as the intervention effect increased and the correlation between baseline and endline measurements decreased at later timepoints.

### 3.2 Imbalanced Scenario: Trials with baseline imbalance

**Figure 3:**
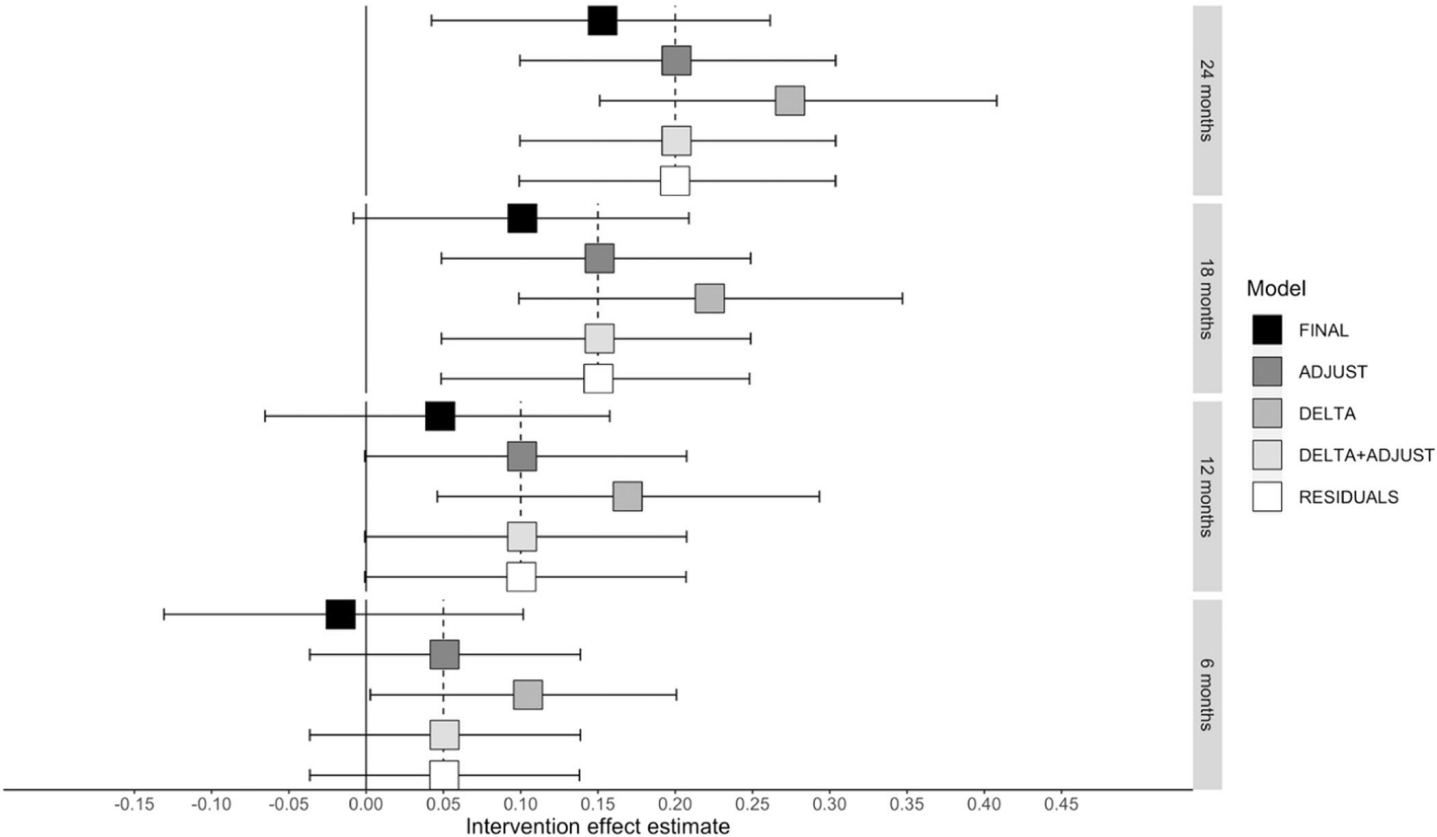
Imbalanced Scenario Effect estimate and 95% uncertainty interval (95% UI) by time point and model when sample size = 1000. Dashed line indicates simulated intervention effect. Boxes not on the dashed line indicates a biased result.

The simulated baseline imbalance resulted in a 0.11 to 0.13 lower mean LAZ in the intervention compared to the control group. This difference was only statistically significant in 8% of trials at the lowest sample size, *n* = 100, but were significantly different in at least 40% of trials with a sample size of 800 or higher. When there was an imbalance in LAZ at baseline, the estimated intervention effects from the FINAL and DELTA methods were systematically biased, whereas the estimates from the ADJUST, DELTA+ADJUST and RESIDUALS method remained unbiased. Figure 3 shows the direction of bias in FINAL and DELTA when the baseline imbalance was in the opposite direction of the intervention effect, demonstrating that the FINAL model underestimated, whereas the DELTA model overestimated, the true intervention effect. ADJUST, DELTA+ADJUST and RESIDUALS produced unbiased estimates at all timepoints. Overall, the absolute value of the bias was greatest for FINAL at the 6‐month timepoint where there was a large correlation between the baseline and the outcome measurements. At 6 months, FINAL underestimated the intervention effect by 140% and reversed the effect on to the other side of the null (−0.02 vs. 0.05). The bias for DELTA was greatest at the 24‐month timepoint where the method overestimated the intervention effect by 35% (0.27 vs. 0.20). The direction of the bias was reversed when the baseline imbalance was in the direction of the intervention effect (Supporting Information: Table S4 and Figure S2).

Coverage in the Imbalanced Scenario, was related to the absolute value of the bias (Supporting Information: Figure S3). As the bias drew closer to zero for FINAL and DELTA, the coverage increased, however it remained between 81% and 89%, lower than the expected 95%. Coverage remained near 95% for ADJUST at all timepoints. The power of ADJUST, DELTA+ADJUST and RESIDUALS in the Imbalanced Scenario (Table 2) was similar to that in the Balanced Scenario (Supporting Information: Table S2). Hence, a baseline imbalance did not impact power when it was appropriately accounted for. Baseline imbalance did not affect mean SE.

**Table 2:** Imbalanced scenario: Model performance by time point for 1000 trials with 1000 children and intervention group mean LAZ 0.12 lower at baseline than control.

Final time point	Model	Simulated effect	Median effect estimate (95% UI)	Bias	Mean SE	Power	Coverage
6 m	FINAL	0.05	−0.02 (−0.13, 0.10)	−0.07	0.06	0.07	0.81
6 m	ADJUST	0.05	0.05 (−0.04, 0.14)	0.00	0.05	0.17	0.96
6 m	DELTA	0.05	0.10 (0.00, 0.20)	0.05	0.05	0.50	0.84
6 m	DELTA+ADJUST	0.05	0.05 (−0.04, 0.14)	0.00	0.05	0.17	0.96
6 m	RESIDUALS	0.05	0.05 (−0.04, 0.14)	0.00	0.05	0.17	0.96
12 m	FINAL	0.10	0.05 (−0.07, 0.16)	−0.05	0.06	0.13	0.86
12 m	ADJUST	0.10	0.10 (0.00, 0.21)	0.00	0.05	0.49	0.95
12 m	DELTA	0.10	0.17 (0.05, 0.29)	0.07	0.06	0.77	0.81
12 m	DELTA+ADJUST	0.10	0.10 (0.00, 0.21)	0.00	0.05	0.49	0.95
12 m	RESIDUALS	0.10	0.10 (0.00, 0.21)	0.00	0.05	0.49	0.95
18 m	FINAL	0.15	0.10 (−0.01, 0.21)	−0.05	0.06	0.41	0.87
18 m	ADJUST	0.15	0.15 (0.05, 0.25)	0.00	0.05	0.84	0.95
18 m	DELTA	0.15	0.22 (0.10, 0.35)	0.07	0.06	0.94	0.8
18 m	DELTA+ADJUST	0.15	0.15 (0.05, 0.25)	0.00	0.05	0.84	0.95
18 m	RESIDUALS	0.15	0.15 (0.05, 0.25)	0.00	0.05	0.84	0.95
24 m	FINAL	0.20	0.15 (0.04, 0.26)	−0.05	0.06	0.77	0.89
24 m	ADJUST	0.20	0.20 (0.10, 0.30)	0.00	0.05	0.97	0.95
24 m	DELTA	0.20	0.27 (0.15, 0.41)	0.07	0.06	0.99	0.8
24 m	DELTA+ADJUST	0.20	0.20 (0.10, 0.30)	0.00	0.05	0.97	0.95
24 m	RESIDUALS	0.20	0.20 (0.10, 0.30)	0.00	0.05	0.97	0.95

*Note*: Bias, average deviation between the effect estimate and the true intervention effect; Coverage, proportion of times the 95% confidence interval contains the true intervention effect 46% trials of 1000 sample size had a statistically significant imbalance at baseline; Median effect estimate, 95% UI (2.5th percentile, 97.5th percentile).

Abbreviations: 95% UI, 95% uncertainty interval; ADJUST, adjusted for baseline; DELTA, change in the measurement over time; DELTA+ADJUST, adjusting for baseline when comparing the changes over time; FINAL, final measurements only; RESIDUAL, baseline in two‐step residuals approach.

ADJUST, DELTA+ADJUST and RESIDUALS produced identical results in terms of bias, power and precision in both the balanced and imbalanced scenarios.

## 4 DISCUSSION

We compared several common strategies used to analyse randomized controlled trial data with a continuous outcome that is also measured at baseline across two illustrative scenarios based on real linear growth study data. ADJUST, DELTA+ADJUST and RESIDUALS all offered the most power across timepoints and sample sizes when there was no baseline imbalance, illustrating principles previously reported in statistical literature (Frison & Pocock, 1992; Fu & Holmer, 2015; Senn, 1994, 2006; Van Breukelen, 2006). The adjusted models also avoided biases seen in estimates from FINAL and DELTA models when there was a baseline imbalance. ADJUST, DELTA+ADJUST and RESIDUALS are mathematically identical and transformations of one another, only differing in the coefficient and the interpretation for baseline LAZ. Although the value and interpretation of the intervention effect are the same, ADJUST model has an advantage over DELTA+ADJUST and RESIDUALS in ease of the baseline coefficient interpretation. These results suggest that ADJUST is the best method to choose for the a priori statistical analysis plan in a linear growth randomized controlled trial.

Despite the benefits to ADJUST, DELTA+ADJUST and RESIDUALS, linear growth randomized trials have frequently not taken advantage of these methods (see example of Mangani et al., 2015 and Hill et al., 2020, which used the DELTA method, and Maleta et al., 2004 and DeBoer et al., 2021, which used the FINAL method; Ren et al., 2022). On face value, each of the five analytic methods, FINAL, ADJUST, DELTA, DELTA+ADJUST and RESIDUALS, appear as reasonable options. The traditional principles of randomized controlled trials suggest the FINAL model is the simplest sufficient analysis, assuming randomization balances out the groups at baseline. The DELTA method appears particularly attractive as it isolates the intervention effect by comparing only what occurred after baseline. However, these approaches increase the sample size necessary to detect intervention effects and ultimately can introduce bias into unbalanced trials. Research offering mathematical proofs and practical validations of these principles, and supporting the use of adjusted models (ADJUST, DELTA+ADJUST or RESIDUALS), are widely available in statistical literature (Egbewale et al., 2014; Frison & Pocock, 1992; Fu & Holmer, 2015; Kahan et al., 2014; Van Breukelen, 2006; Vickers & Altman, 2001).

Adjusted models are known to produce unbiased estimates of the intervention effect in imbalanced trials (Senn, 1994, 2006; Van Breukelen, 2006; Vickers, 2001; Vickers & Altman, 2001). It is a common misconception, known as Lord's paradox (Lord, 1967), that ADJUST and DELTA will produce the same results if there is an imbalance at baseline. Although all methods incorporate the baseline measurement, the DELTA method results in a more restricted model. Essentially, the DELTA method forces the coefficient of the baseline measurement to be 1, whereas the ADJUST method allows the coefficient for the baseline measurement to be estimated based on the data (Van Breukelen, 2006). The inclusion of the baseline measurement allows the adjusted models to account for an imbalance at baseline with no reduction in power, no bias and no loss of precision. The magnitude and the direction of the bias of FINAL and DELTA depend on the direction of the imbalance. When the baseline imbalance is in the opposite direction as the intervention effect, DELTA overestimates the effect with positive bias and FINAL underestimates the effect with negative bias. Other studies show the magnitude of this bias increases with the baseline imbalance and the correlation between baseline and endpoint measurements (Egbewale et al., 2014).

The performance of the DELTA and FINAL models were highly influenced by the correlation between baseline and the endpoint measurements (Egbewale et al., 2014; Frison & Pocock, 1992; Vickers, 2001). When correlation is more than about 0.5, as should be expected for LAZ trials with 6 months follow‐up or less, DELTA has more power and lower SE than FINAL. This relationship is reversed and FINAL has more power and lower SE than DELTA, when the correlation is less than about 0.5 as may be seen with more distal endpoints (e.g., at 12, 18 and 24 months). In the context of randomized controlled trials for a linear growth outcome, the time between baseline and the endpoint is the primary driver of correlation, and thus the power of these methods. As seen in MAL‐ED, measurements closer together (e.g., 0 and 6 months) had higher correlation than measurements farther apart (e.g., 0 and 24 months). The correlation between two time points may also be influenced by the age of the children and will be lower during periods of rapid linear growth. Other continuous outcomes like weight or developmental scores may have even lower correlation over time. In all randomized settings, ADJUST, DELTA+ADJUST and RESIDUALS offer increased power and lower SE, although the benefit over DELTA or FINAL methods may be marginal when the correlation is very high or very low, respectively (Vickers, 2001; Vickers & Altman, 2001).

The main limitation of ADJUST is that it relies on using linear regression, which is subject to the assumption that the outcome is normally distributed. FINAL may be preferred to limit the distributional assumptions required, as a simple *t* test can be used instead of a regression model. However, the substantial gains in bias and precision afforded by the ADJUST, DELTA+ADJUST and RESIDUALS methods likely outweigh this consideration when baseline measurements are available and, especially, when they may be imbalanced. The substantially increased sample size required to perform an informative *t* test compared with adjusted regression analysis will unnecessarily expose additional study participants to an unproven intervention and inflate the costs of the trial.

This study simulated linear growth and correlation parameters based on a well‐known birth cohort, MAL‐ED, and the intervention effect is based on the SHINE trial. The selection of data sources were based, in part, by the author's familiarity and access, however, they provided realistic parameters for the simulations including, intervention effect, correlation between timepoints and average LAZ at timepoints. These results apply to a number of randomized trials that assessed linear growth as the primary outcome and were powered for similar effect sizes (Deboer et al., 2018; The ABCD Study Team, 2020). The baseline imbalance simulated in this study was fairly large for illustrative purposes and may be frequently smaller in large clinical trials. However, given that analysis plans are made before the trial, investigators should still have a written analysis plan in provision to protect against imbalance.

This study stimulated a birth cohort where the intervention and randomization occur at birth and the primary outcome is a single postintervention measurement in the context of a randomized trial. However, the same principles described here could be applied to other scenarios, including observational cohort studies where confounding (i.e., baseline imbalance) is expected, and other growth outcomes such as weight‐for‐age *z*‐score, weight‐for‐height *z*‐score, mid‐upper arm circumference (MUAC) *z*‐score or even absolute measures of weight, height, MUAC and LAZ difference. Extensions of the analyses explored here are also possible, for example, incorporating multiple postrandomization endpoints using linear mixed effects models. Of note, the analytic recommendation for pregnancy studies where intervention occurs before birth may differ considering the birth measurement is not at baseline but is an intermediate measurement after randomization and therefore may be affected by the intervention (a causal intermediate).

## 5 CONCLUSION

In summary, statistical methods adjusting for baseline, ADJUST, is the recommended analytic approach for randomized controlled trials assessing the effect of an intervention on a linear growth outcome between baseline and a single postintervention measurement. ADJUST, DELTA+ADJUST and RESIDUALS offered more power and precision compared with the other methods assessed, and ADJUST provides easier interpretability over DELTA+ADJUST and RESIDUALS. These results warn against using FINAL or DELTA as they offer suboptimal power and baseline imbalances in the outcome or other potential confounding factors may result in biased results.

## AUTHOR CONTRIBUTIONS

The analysis was done by Emily L. Deichsel. Emily L. Deichsel, Kirkby D. Tickell and Elizabeth T. Rogawski McQuade participated in design of the analysis and writing of the manuscript.

## CONFLICT OF INTEREST STATEMENT

The authors declare no conflict of interest.

## Supporting information

Supporting Information.

## Data Availability

The data that support the findings of this study are available in the Supporting Information: Material of this article.
