# Supplementary material for: Minimizing error in estimates of the effect of interventions by accounting for baseline measurements: A simulation study analyzing effects on child growth
Source: Matern Child Nutr. 2023 Jul 13;19(4):e13547. doi: 10.1111/mcn.13547 (PMC10483953; doi:10.1111/mcn.13547)
Supplement: Supplementary file 1 — Supporting Information. [file MCN-19-e13547-s001.docx]

**Supplementary Materials**

**Table S1** Mean imbalance between Intervention groups in the Balanced and Imbalanced Scenarios.

| **Sample size** | **Mean imbalance** | **Percent trials with statistically significant imbalance** |
| --- | --- | --- |
| Balanced Scenario | | |
| 100 | 0.01 | 5% |
| 200 | 0.01 | 6% |
| 300 | 0.00 | 6% |
| 400 | 0.00 | 5% |
| 500 | 0.00 | 5% |
| 600 | 0.00 | 4% |
| 700 | -0.01 | 5% |
| 800 | 0.00 | 5% |
| 900 | 0.00 | 6% |
| 1000 | 0.00 | 5% |
| 1100 | 0.00 | 3% |
| 1200 | 0.00 | 5% |
| 1300 | 0.00 | 5% |
| 1400 | 0.00 | 6% |
| 1500 | 0.00 | 5% |
| Imbalanced scenario | | |
| 100 | -0.11 | 8% |
| 200 | -0.12 | 12% |
| 300 | -0.12 | 16% |
| 400 | -0.13 | 24% |
| 500 | -0.12 | 26% |
| 600 | -0.12 | 32% |
| 700 | -0.13 | 38% |
| 800 | -0.12 | 41% |
| 900 | -0.12 | 46% |
| 1000 | -0.12 | 46% |
| 1100 | -0.12 | 54% |
| 1200 | -0.12 | 55% |
| 1300 | -0.12 | 59% |
| 1400 | -0.12 | 64% |
| 1500 | -0.12 | 66% |

**Table S2** Balanced Scenario: Model performance by timepoint for 1000 trials with 1000 children and no baseline imbalance in LAZ

| **Final timepoint** | **Model** | **Intervention effect** | **Median effect estimate (95% UI)**  **(2.5^th^ pct, 97.5th pct)** | **Bias** | **Mean Standard error** | **Power** | **Coverage** |
| --- | --- | --- | --- | --- | --- | --- | --- |
| 6m | FINAL | 0.05 | 0.05 (-0.06, 0.16) | 0.00 | 0.06 | 0.14 | 0.96 |
| 6m | ADJUST | 0.05 | 0.05 (-0.04, 0.14) | 0.00 | 0.05 | 0.19 | 0.95 |
| 6m | DELTA | 0.05 | 0.05 (-0.06, 0.16) | 0.00 | 0.05 | 0.15 | 0.94 |
| 6m | DELTA+ADJUST | 0.05 | 0.05 (-0.04, 0.14) | 0.00 | 0.05 | 0.19 | 0.95 |
| 6m | RESIDUALS | 0.05 | 0.05 (-0.04, 0.14) | 0.00 | 0.05 | 0.19 | 0.95 |
| 12m | FINAL | 0.10 | 0.10 (-0.01, 0.22) | 0.00 | 0.06 | 0.39 | 0.94 |
| 12m | ADJUST | 0.10 | 0.10 (0.00, 0.21) | 0.00 | 0.05 | 0.47 | 0.95 |
| 12m | DELTA | 0.10 | 0.10 (-0.02, 0.22) | 0.00 | 0.06 | 0.36 | 0.95 |
| 12m | DELTA+ADJUST | 0.10 | 0.10 (0.00, 0.21) | 0.00 | 0.05 | 0.47 | 0.95 |
| 12m | RESIDUALS | 0.10 | 0.10 (0.00, 0.21) | 0.00 | 0.05 | 0.47 | 0.95 |
| 18m | FINAL | 0.15 | 0.15 (0.04, 0.27) | 0.00 | 0.06 | 0.73 | 0.94 |
| 18m | ADJUST | 0.15 | 0.15 (0.05, 0.26) | 0.00 | 0.05 | 0.83 | 0.94 |
| 18m | DELTA | 0.15 | 0.15 (0.03, 0.28) | 0.00 | 0.06 | 0.64 | 0.96 |
| 18m | DELTA+ADJUST | 0.15 | 0.15 (0.05, 0.26) | 0.00 | 0.05 | 0.83 | 0.94 |
| 18m | RESIDUALS | 0.15 | 0.15 (0.05, 0.26) | 0.00 | 0.05 | 0.83 | 0.94 |
| 24m | FINAL | 0.20 | 0.20 (0.09, 0.31) | 0.00 | 0.06 | 0.93 | 0.95 |
| 24m | ADJUST | 0.20 | 0.20 (0.10, 0.31) | 0.00 | 0.05 | 0.97 | 0.95 |
| 24m | DELTA | 0.20 | 0.20 (0.08, 0.33) | 0.00 | 0.06 | 0.86 | 0.95 |
| 24m | DELTA+ADJUST | 0.20 | 0.20 (0.10, 0.31) | 0.00 | 0.05 | 0.97 | 0.95 |
| 24m | RESIDUALS | 0.20 | 0.20 (0.10, 0.31) | 0.00 | 0.05 | 0.97 | 0.95 |

Bias: average deviation between the effect estimate and the true intervention effect

Power: proportion of the times a false null hypothesis is rejected

Coverage: proportion of times the 95% confidence interval contains the true intervention effect

**Table S3**: Sample size required to meet 80% power, assuming no baseline imbalance

| **Final timepoint** | **Model** | **Sample size** | **Power** |
| --- | --- | --- | --- |
| 6m | FINAL | 9300 | 0.80 |
| 6m | ADJUST | 6300 | 0.81 |
| 6m | DELTA | 8300 | 0.82 |
| 6m | DELTA+ADJUST | 6300 | 0.81 |
| 6m | RESIDUALS | 6300 | 0.81 |
| 12m | FINAL | 2800 | 0.82 |
| 12m | ADJUST | 1200 | 0.81 |
| 12m | DELTA | 3000 | 0.81 |
| 12m | DELTA+ADJUST | 1200 | 0.81 |
| 12m | RESIDUALS | 1200 | 0.81 |
| 18m | FINAL | 1200 | 0.81 |
| 18m | ADJUST | 900 | 0.81 |
| 18m | DELTA | 1500 | 0.81 |
| 18m | DELTA+ADJUST | 900 | 0.81 |
| 18m | RESIDUALS | 900 | 0.81 |
| 24m | FINAL | 700 | 0.81 |
| 24m | ADJUST | 600 | 0.84 |
| 24m | DELTA | 900 | 0.84 |
| 24m | DELTA+ADJUST | 600 | 0.84 |
| 24m | RESIDUALS | 600 | 0.84 |

**Table S4**: Model performance by timepoint 1000 sample size. Baseline imbalance reverse, control group mean 0.12 lower LAZ at baseline than control.

| **Final timepoint** | **Model** | **Simulated effect** | **Median effect estimate (95% UI)** | **Bias** | **Mean Standard error** | **Power** | **Coverage** |
| --- | --- | --- | --- | --- | --- | --- | --- |
| 6m | FINAL | 0.05 | 0.12 (0.00, 0.23) | 0.07 | 0.06 | 0.51 | 0.81 |
| 6m | ADJUST | 0.05 | 0.05 (-0.04, 0.14) | 0.00 | 0.05 | 0.17 | 0.96 |
| 6m | DELTA | 0.05 | 0.00 (-0.10, 0.10) | -0.05 | 0.05 | 0.04 | 0.84 |
| 6m | DELTA+ADJUST | 0.05 | 0.05 (-0.04, 0.14) | 0.00 | 0.05 | 0.17 | 0.96 |
| 6m | RESIDUALS | 0.05 | 0.05 (-0.04, 0.14) | 0.00 | 0.05 | 0.17 | 0.96 |
| 12m | FINAL | 0.10 | 0.15 (0.04, 0.27) | 0.05 | 0.06 | 0.75 | 0.87 |
| 12m | ADJUST | 0.10 | 0.10 (-0.01, 0.20) | 0.00 | 0.05 | 0.48 | 0.95 |
| 12m | DELTA | 0.10 | 0.03 (-0.09, 0.15) | -0.07 | 0.06 | 0.07 | 0.81 |
| 12m | DELTA+ADJUST | 0.10 | 0.10 (-0.01, 0.20) | 0.00 | 0.05 | 0.48 | 0.95 |
| 12m | RESIDUALS | 0.10 | 0.10 (-0.01, 0.20) | 0.00 | 0.05 | 0.48 | 0.95 |
| 18m | FINAL | 0.15 | 0.20 (0.09, 0.31) | 0.05 | 0.06 | 0.94 | 0.87 |
| 18m | ADJUST | 0.15 | 0.15 (0.05, 0.25) | 0.00 | 0.05 | 0.85 | 0.95 |
| 18m | DELTA | 0.15 | 0.08 (-0.05, 0.2) | -0.07 | 0.06 | 0.23 | 0.8 |
| 18m | DELTA+ADJUST | 0.15 | 0.15 (0.05, 0.25) | 0.00 | 0.05 | 0.85 | 0.95 |
| 18m | RESIDUALS | 0.15 | 0.15 (0.05, 0.25) | 0.00 | 0.05 | 0.85 | 0.95 |
| 24m | FINAL | 0.20 | 0.25 (0.14, 0.36) | 0.05 | 0.06 | 0.99 | 0.89 |
| 24m | ADJUST | 0.20 | 0.20 (0.10, 0.30) | 0.00 | 0.05 | 0.97 | 0.95 |
| 24m | DELTA | 0.20 | 0.13 (-0.01, 0.25) | -0.07 | 0.06 | 0.51 | 0.8 |
| 24m | DELTA+ADJUST | 0.20 | 0.20 (0.10, 0.30) | 0.00 | 0.05 | 0.97 | 0.95 |
| 24m | RESIDUALS | 0.20 | 0.20 (0.10, 0.30) | 0.00 | 0.05 | 0.97 | 0.95 |

Median effect estimate: 95% UI (2.5^th^ pct, 97.5^th^ pct)

Bias: average deviation between the estimate and the truth

Coverage: proportion of times the 95% confidence interval contains the true value


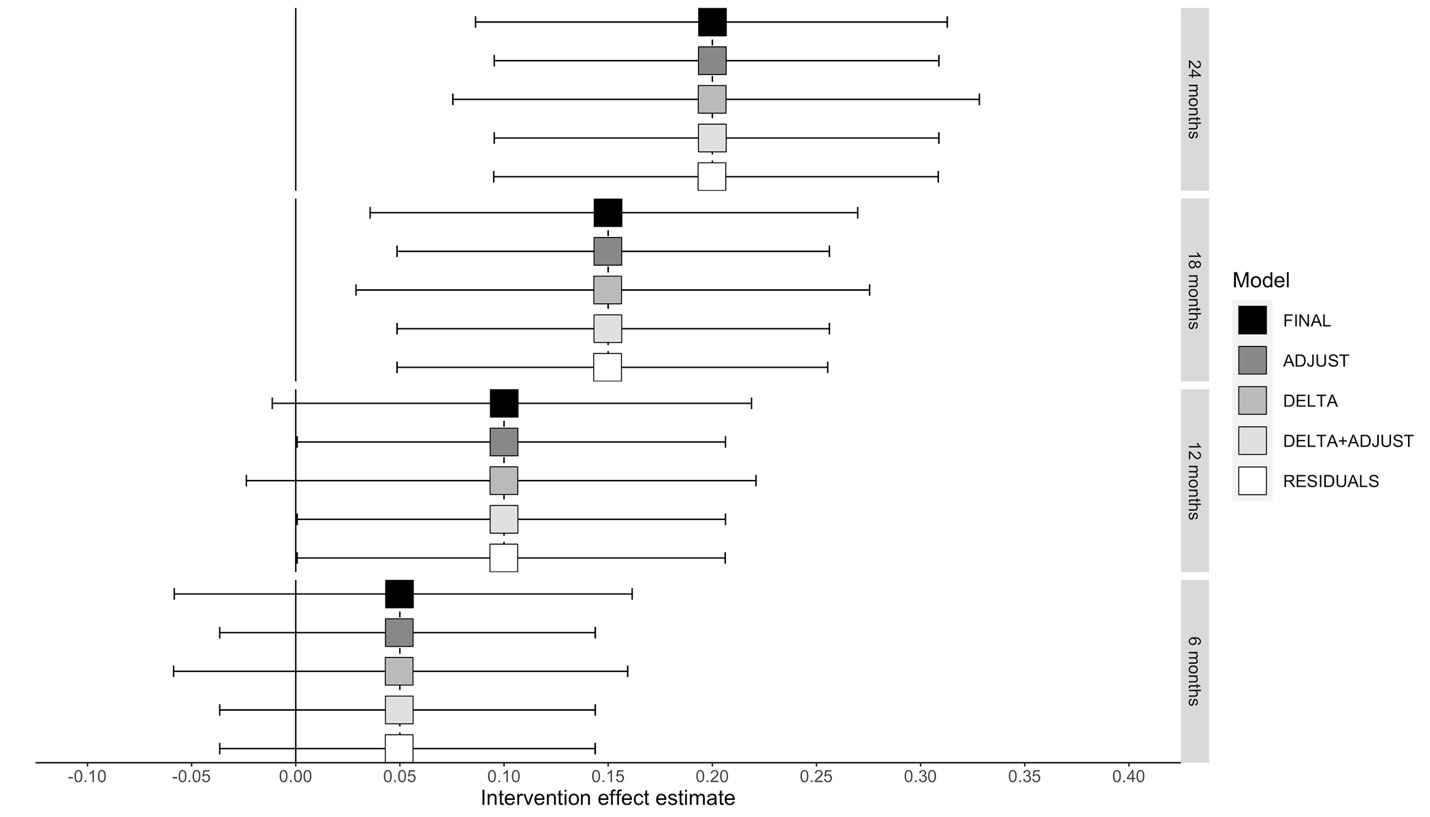


**Figure S1:** Balanced Scenario effect estimate and 95% UI by time point and model method when sample size=1000. Dashed line indicates simulated intervention effect.


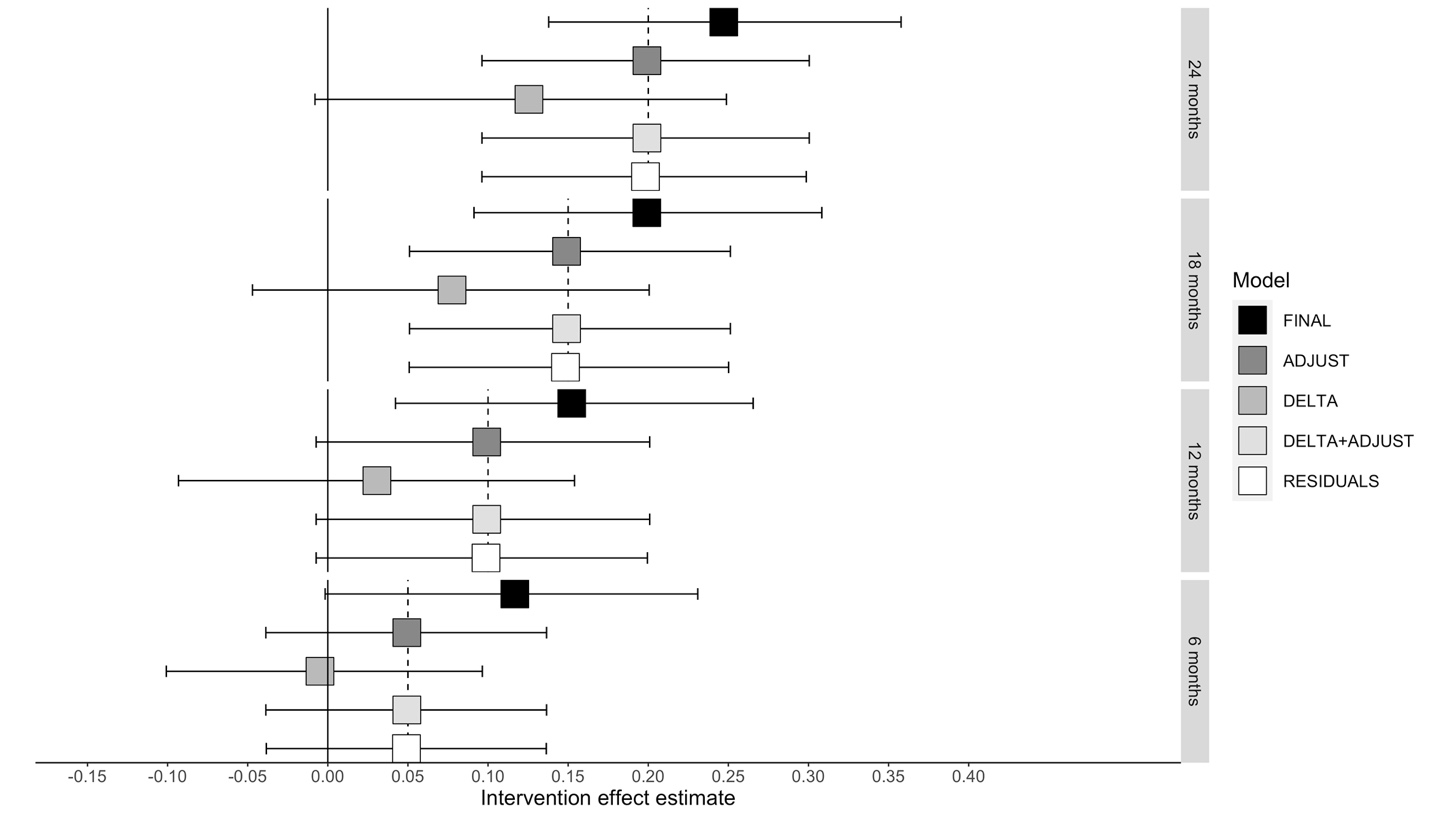


**Figure S2:** Effect estimate and 95% UI by time point and model method when sample size=1000. Baseline Imbalance reverse, control group mean 0.12 lower LAZ at baseline than control. Boxes not on the dashed line indicates a biased result.


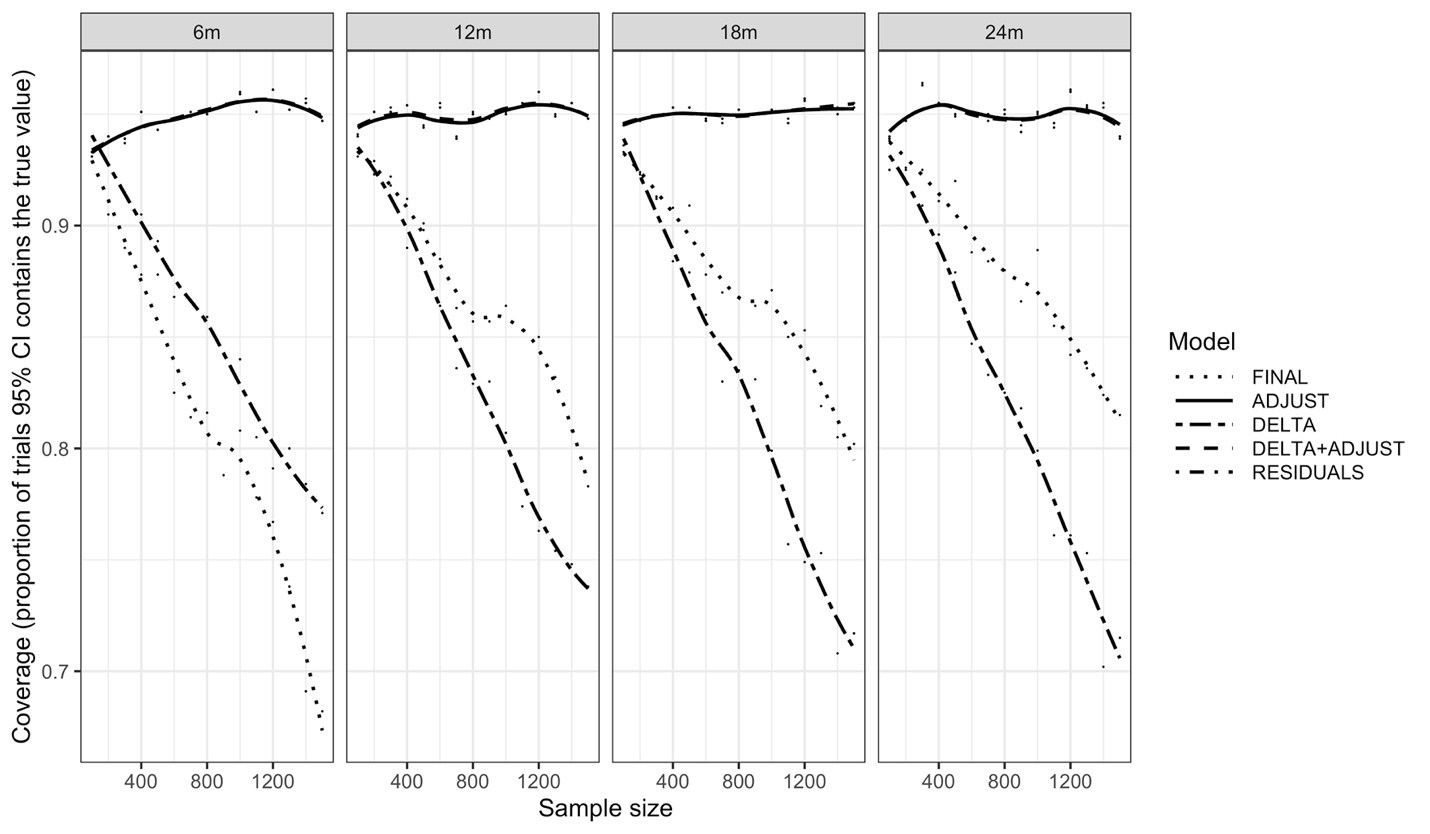


**Figure S3:** Imbalanced baseline: coverage of 95% UI (the proportion of trial 95% UIs that contain the true effect) at each timepoint by model and sample size (lowess). Low coverage indicates bias in the result. DELTA+ADJUST and RESIDUALS model results are identical to ADJUST model result and so lines overlap

**Methods**

For the simulation for the Unbalanced Scenario, the probability of being in the intervention group was 0.47-0.03*baseline LAZ, such that a lower birth LAZ increased the probability of being in the intervention group. This allocation induced a baseline imbalance where the mean LAZ was lower in the intervention group than the control group but maintained an allocation ratio close to 1:1.

**Code**

#Simulation program in R (R Studio) using parameters from MAL-ED Table 1. The prime identifies values that are specific to particular simulation. samplesize’ indicates samplesize=100, samplesize=200, samplesize=300, samplesize=400, … samplesize=1400, samplesize=1500. The asterisk represents repeated for each timepoint m* indicates timepoint=6m, timepoint=12m, timepoint=18m, timepoint=24m.

library(dplyr)

library(tidyr)

library(faux)

mmat<-(-1.10, -1.19, -1.66, -1.95, -2.03)

sdmat<-(1.03, 0.97, 0.93, 0.93, 0.94)

cmat.data<-(1, 0.61, 0.47, 0.46, 0.44,

0.61, 1, 0.87, 0.86, 0.76,

0.47, 0.87, 1, 0.90, 0.88,

0.46, 0.80, 0.9, 1, 0.95,

0.44, 0.76, 0.88, 0.95, 1)

cmat<- matrix(cmat.data, nrow=5)

df<-rnorm_multi(samplesize’, 5, mu=mmat, sd=sdmat, r=cmat, varnames=c("m0", "m6", "m12", "m18", "m24"))

#*generates a single birth cohort of particular sample size based on MAL-ED parameters*

n=1

#*sets normal distribution for intervention group randomization*

p=0.5

#*sets probability of being in intervention group. Unbalanced Baseline scenario uses p=0.47-0.03*df$m0, the reverse Unbalanced Baseline scenario uses p=0.53+0.03*df$m0*

df$intervention<-rbinom(nrow(df), n, p)

#*identifies two treatment groups – Control (0); Treatment (1); 1:1* *allocation*

df$birth_laz<-df$m0

#*renames baseline value*

df$m6<-ifelse(df$intervention==1, df$m6+0.05, df$m6)

#*applies 0.05 intervention effect in intervention group at 6 month timepoint*

df$m12<- ifelse(df$intervention==1, df$m12+0.1, df$m12)

#*applies 0.1 intervention effect in intervention group at 12 month timepoint*

df$m18<- ifelse(df$intervention==1, df$m18+0.15, df$m18)

#*applies 0.15 intervention effect in intervention group at 18 month timepoint*

df$m24<-ifelse(df$intervention==1, df$m24+0.2, df$m24)

#*applies 0.2 intervention effect in intervention group at 24 month timepoint*

lm(m*~intervention, data=df)

#*performs FINAL analysis at each timepoint*

lm(m*_laz~intervention + birth_laz, data=df)

#*performs ADJUST analysis* *at each timepoint*

df$delta_m*<-(df$m*-df$birth_laz)

#*generates changes score for the treatment groups at each timepoint*

lm(delta_m*~intervention, data=df)

#*performs DELTA analysis at each timepoint*

lm(delta_m*~intervention + birth_laz, data=df)

#*performs DELTA+ADJUST analysis at each timepoint*

residM1<-resid((lm(m*~birth_laz), data=df))

#*step one of residual method*: *generates adjusted residuals at each timepoint*

lm(residM1~intervention, data=df)

#*step two of residual method: estimates difference in residuals for the treatment groups at each timepoint*
